# Cubosome Based Ion-Selective Optodes–Toward Tunable Biocompatible Sensors

**DOI:** 10.1021/acs.analchem.1c01247

**Published:** 2021-09-21

**Authors:** Emilia Stelmach, Ewa Nazaruk, Krzysztof Maksymiuk, Agata Michalska

**Affiliations:** Faculty of Chemistry, University of Warsaw, Pasteura 1, 02-093 Warsaw, Poland

## Abstract

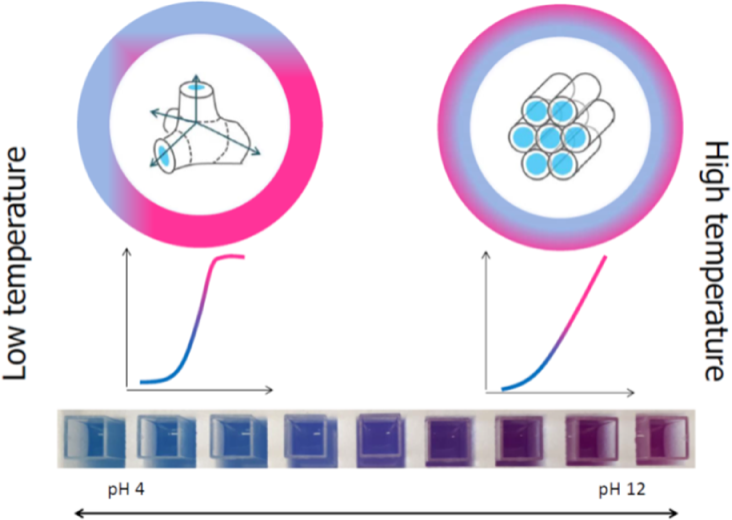

We report here on a new generation of optical ion-selective sensors benefiting from cubosomes or hexosomes–nanostructural lipid liquid phase. Cubosome as well as hexosome optodes offer biocompatibility, self-assembly preparation, high stability in solution, and unique, tunable analytical performance. The temperature trigger reversibly changes the lipid nanoparticle internal structure–changing analyte access to the bulk of the probe and ultimately affecting the response pattern. Thus, cubosome or hexosome optodes are highly promising alternatives to conventional polymeric based optical nanoprobes.

Optodes, benefiting from highly selective ionophores, allow optical insight into ion concentration changes.^1^ The probes evolved from polymeric films^2^ to nanostructures.^3−5^ Bulk reaction of the optode with the analyte results in high sensitivity signals covering a relatively narrow concentration range, whereas confinement of the reaction zone to the surface most layer leads to linear dependence of emission recorded on logarithm of concentration covering a few orders of magnitude range.^6^

Optodes, similar to other polymeric ion-selective sensors,^1−4^ require the presence of a plasticizer–toxic organic liquids,^7,8^ most often bis(2-ethylhexyl) sebacate as the minor^9^ or major^10,11^ constituent. The presence of a plasticizer^7,8^ is clearly a bottleneck of application in a real analytical scenario. The spontaneous release of a plasticizer, increased in the presence of an ionophore and ion-exchanger,^12^ gives rise to severe toxicity hazards.^7,8^

To advance nanoptode sensors and to make these devices safe for traditional biomedical as well as new applications, an alternative approach is required. In this work, lipid based nanoptodes are proposed. The choice of lipids as sensor matrix material is justified primarily by proven biocompatibility. This allows application of nanostructural lipid systems–cubosomes or hexosomes–as contrast agents,^13^ drugs,^14−16^ or protein^17,18^ carriers. The biocompatibility and low toxicity of lipids, e.g., phytantriol, have been generally accepted for many years now.^19^

Moreover, stable in solution cubosomes/hexosomes are obtained in a simple process. Surprisingly, lipid nanostructures were not of interest as ion-selective sensor matrices before^20,21^ although biosensing with these systems has been considered.^22^

From the point of view of ion-sensing, the additional advantage has the possibility of adjusting the internal arrangement of channels by a temperature trigger, affecting accessibility of the bulk probe for the analyte and ultimately controlling the response pattern of the sensor. Cubosomes consist of two interpenetrating, noncontacting aqueous channels that are surrounded by a lipid bilayer arranged in a thermodynamically favorable periodic 3D structure.^20,23,24^ The channel system open toward the sample (cubic phase, (V_2_)) allows penetration of the bulk structure, thus sigmoidal shape dependence of signal vs analyte concentration is expected. The closed system (hexagonal phase, (H_2_)), hexosomes, consists of closed reverse micellar cylinders that are arranged in a 2D hexagonal lattice^25^ that allows only the nanoparticle surface to be in contact with the sample offering linear dependence of signal vs logarithm of analyte concentration. Different additives needed to render lipid nanostructure ion-selectivity can affect the structure of the resulting sensors, offering various analytical advantages.

Phytantriol (PT) is used as a model lipid in forming the *Pn*3̅*m* cubic phase in a water environment at room temperature^25^ and undergoes transition to a H_2_ phase when heated to ca. 44 °C. This, in principle, offers the unique possibility to adjust the response pattern of cubosome/hexosome optodes using a temperature trigger. If the sample and the probe are kept above 44 °C, a linear dependence is expected, whereas for lower temperatures, the sigmoidal type relation of signal vs logarithm of concentration will prevail. As model sensors, pH sensitive optodes were studied. Lipid nanostructures tested were prepared according to the procedure developed previously^26^ using chromoionophore I and potassium tetrakis(4-chlorophenyl)borate to result in H^+^-selective optodes or additionally containing calcium-selective ionophore to result in Ca^2+^-selective optodes.

## Experimental Section

### Reagents

Chromoionophore I (*N*-octadecanoyl-Nile blue), potassium tetrakis(4-chlorophenyl)borate (KTChP), calcium ionophore: diethyl *N*,*N*′-[(4*R*,5*R*)-4,5-dimethyl-1,8-dioxo-3,6-dioxaoctamethylene]bis(12-methylaminododecanoate) (ETH 1001), Pluronic F108 (PF108), and monoolein were purchased from Aldrich (Germany). Phytantriol (PT) used for the synthesis of the mesophases was purchased from Tokyo Chemical Industry (TCI).

Doubly distilled and freshly deionized water (resistance 18.2 MΩ cm, Milli-Qplus, Millipore, Austria) was used throughout this work. The following universal pH buffers were used (mixture of 0.109 M citric acid, 0.1 M Tris, 0.088 M NaH_2_PO_4_, and 0.1 M NaCl adjusted with HCl or NaOH to the desired pH values).

### Apparatus

Fluorimetric experiments were performed using a Cary Eclipse spectrofluorimeter (Varian). After exposure at an excitation wavelength of 580 nm, emission intensity was recorded within the range from 600 to 800 nm. Unless otherwise stated, the slits used were 5 nm for both excitation and emission, while the detector voltage was maintained at 800 V.

2D electron cryomicroscopy images were taken on a Thermo Fisher Glacios TEM operating at 200 kV. Cubosome dispersions were plunge-frozen onto Quantifoil R2/2 holey carbon grids using a Thermo Fisher Vitrobot.

Small angle X-ray scattering (SAXS) was performed using a Bruker Nanostar system working with CuKα radiation equipped with a Vantec 2000 area detector. Measurements were performed at 24 and 60 °C; the scattered intensity was collected over 1 h. The 2D pattern was integrated into a 1D scattering function *I*(*q*) (where *q* (nm^–1^) is the length of the scattering vector). To identify the phase type, the scattering vector (*q*) values of the peaks were correlated with Miller indices for known mesophases.

### Optical Measurements

For fluorescence measurements, 3 mL of a sample solution was used: the pH buffer was stabilized in a temperature of 20 or 60 °C for 2 min. After 2 min, 20 μL of cubosome/hexosome suspension was added to the pH buffer or for calcium sensors to a calcium ion buffered solution.

### Cubosome Preparation

Cubosomes were prepared according to the slightly modified procedure given in our previous paper.^26,27^ To prepare H^+^-selective nanostructures, cubosomes were loaded with chromoionophore I and an ion-exchanger (potassium tetrakis(4-chlorophenyl)borate)–1.2 mg of chromoionophore I and 2.3 mg of potassium tetrakis(4-chlorophenyl)borate (KTChP) were mixed with melted phytantriol (PT) (50 mg) (or monoolein in the control experiment) and left for ca. 30 min at 54 °C to obtain a homogeneous sample. The procedure of calcium ionophore containing sensor preparation–Ca^2+^-selective optodes–was the same as that described above. One milliliter of dispersion contained 1.2 mg of chromoionophore I, 2 mg of an ion-exchanger (KTChP), 4 mg of calcium ionophore, and 50 mg of phytantriol. The samples were then hydrated in the presence of a stabilizer, Pluronic F108 (1 mL). The emulsification was conducted using SONICS Vibracell VCX 130 (Sonics & Materials Inc.) at 40% for 20 min (2 s sonic pulses interrupted by 3 s breaks). Prior to use, samples were equilibrated at room temperature for at least 24 h.

## Results and Discussion

### H^+^-Selective Lipid Based Nanostructural Optodes

**Figure 1:**
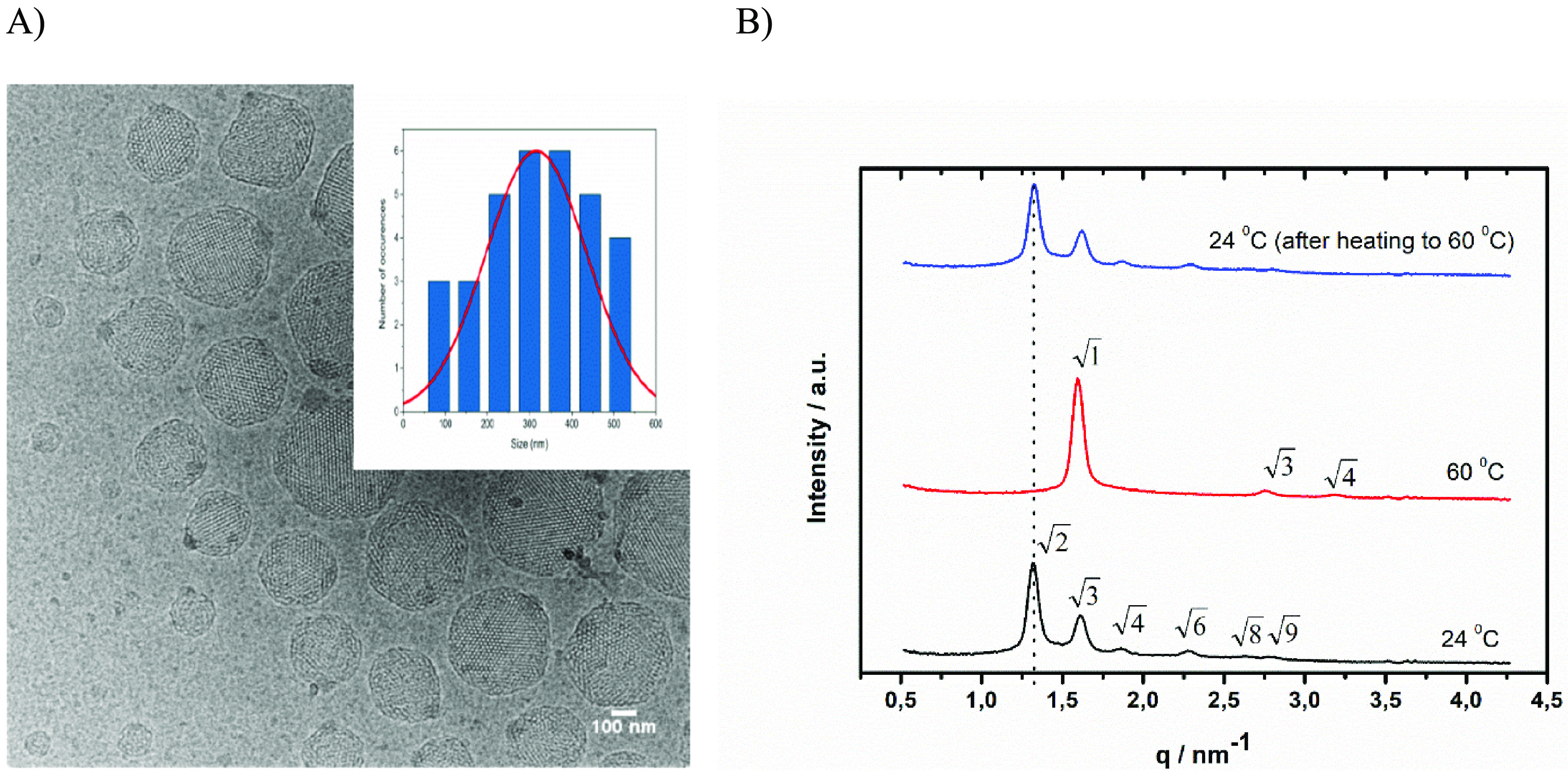
A) Cryogenic transmission electron microscopy (cryo-TEM) images of cubosome optodes and size distribution of obtained structures. B) Representative SAXS diffraction patterns obtained for sensors at 24 °C, 60 °C, and 24 °C following equilibration after heating to 60 °C.

**Figure 2:**
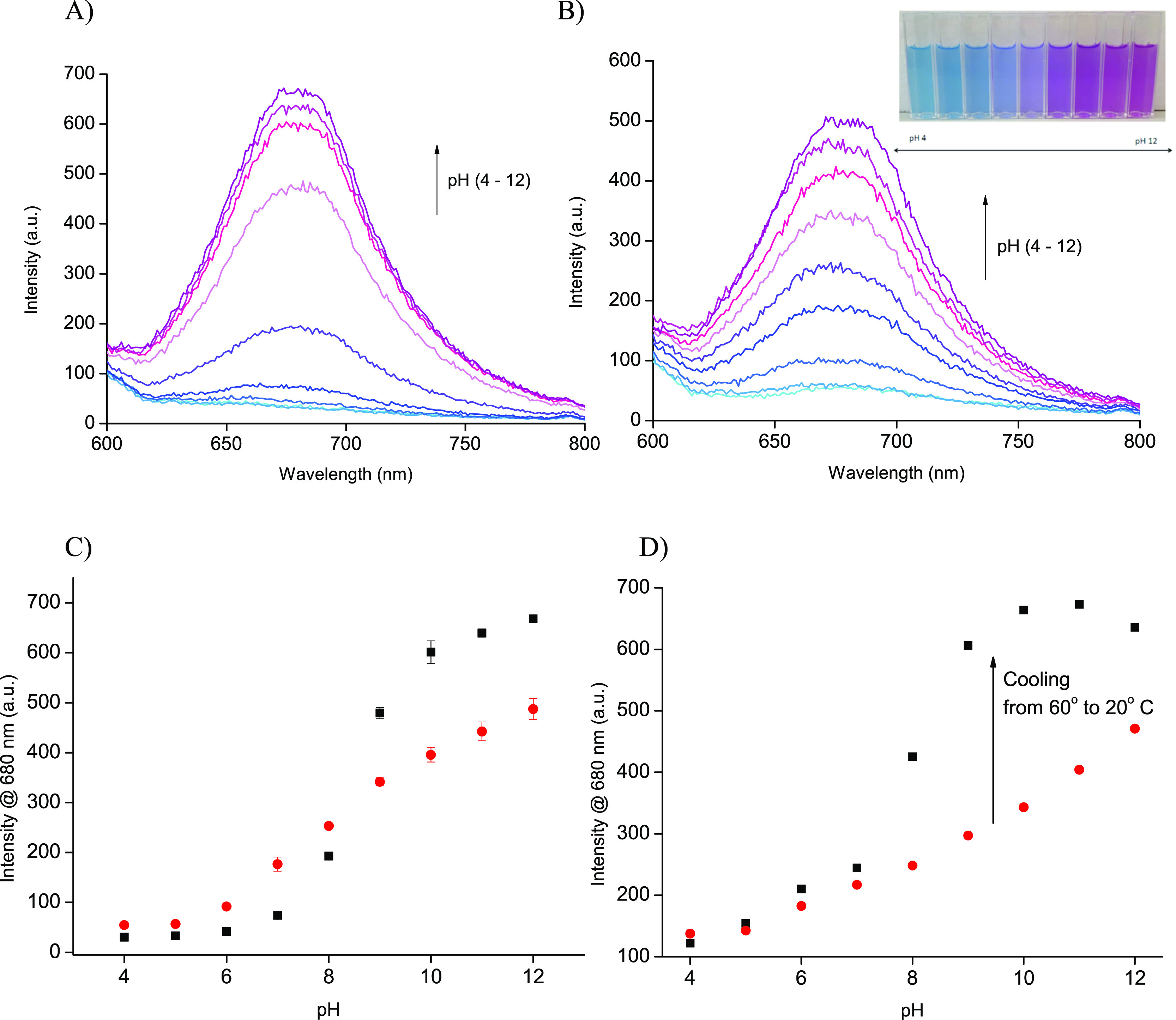
Effect of pH change on emission of cubosome optodes: emission spectra recorded at A) 20 °C and B) 60 °C; emission at maximum at 680 nm dependence on pH recorded for (■) 20 °C or (●, red) 60 °C: C) the mean signal recorded ± the standard deviation from two measurements and D) transition of dependence for applied temperature trigger–cooling from 60 to 20 °C.

The cryo-TEM images obtained, Figure 1A, revealed a well-ordered structure inside the nanoparticles. The mean diameter of prepared cubosomes was close to 300–400 nm. Figure 1B shows representative X-ray diffractograms of prepared nanostructural optodes. The 1D diffraction patterns collected at 24 °C exhibit a sequence of diffraction peaks with relative positions at ratios of √2:√3:√4:√6:√8, which can be attributed to the double diamond (*Pn*3̅*m*) symmetry with a lattice parameter (*a*) of 6.7 nm. Water channels are open toward the sample allowing an exchange of ions and ultimately the bulk reaction of nanoptode. The optical spectra of cubosome optodes pretreated and tested at 20 °C show an increase of emission intensity for the pH increase, Figure 2A and Figure S1. The emission peak is formed at 680 nm, similar to other optode systems.^4,10^ This suggests that the chromophore groups are facing a hydrophilic environment, i.e., are located close to the channel, whereas the alkyl side chain is located in-between lipid layers.^9^ The bulk reaction of the probe resulted in the sigmoidal dependence of emission read at maximum on pH, Figure 2C, with maximum sensitivity between pH 7 and 10. The response time of cubosome nanosensors was below 15 s, i.e., the time required to mix the sample and probes and to start the experiment.

At a temperature of 60 °C, X-ray studies reveal temperature triggered transformation of structures. The three Bragg reflections follow the relationship 1:√3:√4, which corresponds to the hexagonal (*p*6*m*) space group, with a lattice parameter (*a*) of 4.5 nm, Figure 1B. This structure is made up of densely packed water filled cylindrical micelles, and the solution inside the cylindrical micelles is not in contact with the water outside directly.^28^ Thus, the diffusion of the analyte into the bulk optode is hindered; only the outer surface of the optode is able to interact with the sample.

The optical spectra recorded for samples that were prepared at 60 °C are similar to those described above with maximum of emission at 680 nm for alkaline samples, Figure 2B. The emission recorded is plotted against the pH values as determined for used buffers at 24 °C, although the pH decreases with a temperature increase; for the applied buffer this decrease should be lower than one unit. On the other hand, the neutrality point in water at 60 °C shifts to a lower pH, thus both mentioned effects compensate to some extent. This conclusion seems supported by results for monoolein, not undergoing transformation at a higher temperature, where within the range of experimental error similar results were obtained at both temperatures, Figure S2B. The emission (read at 680 nm) plot on pH was linear within the broad range covering pH from 6 to 12 (*R*^2^ = 0.982), Figure 2C. It should be stressed that obtained linear dependence covers 6 orders of magnitude, thus cubosomes offer one of the widest linear response ranges ever reported for pH optodes.^1,4,10,11^

Acknowledging the fact that temperature change results in change of the cubosome internal structure, it is rational to expect trigger (cooling) applied postcontact of probes with the sample will change linear dependence of emission on pH recorded for the hexagonal phase (closed system) to sigmoidal shape dependence for double diamond (open channels) cubosomes. On the other hand, transformation of the *Pn*3̅*m* cubic phase open structure to the H_2_ closed system (heating) will not affect the response pattern.

X-ray studies confirmed that cooling the sample from 60 to 25 °C results in restoration of all reflections characteristic for the *Pn*3̅*m*-cubic double diamond phase with a lattice parameter of 6.7 nm, Figure 1B.

Figure 2D shows calibration recorded for samples initially at 60 °C and then postcooling to 20 °C. A change in the sample temperature resulted in a pronounced change of signal vs pH relation, and an opening of channels allowed a bulk reaction of the cubosome optode, ultimately resulting in transition from linear to sigmoidal type dependence. A pronounced increase in absolute signal intensities accompanying increased sensitivity for the pH range from 7 to 10 is potentially useful for practical applications, allowing simple achieving of a vast increase in sensitivity, by change of sample/probes mixture temperature–the tuning signal, if required, Figure S2A, presents results of the experiment, in which an initially open channel structure was transformed to a closed one by a temperature change from 20 to 60 °C; as expected, no change in the response pattern was observed, Figure S2A. It should be stressed that in a control experiment performed for monoolein structures of a much higher transition temperature (ca. 95 °C) compared to phytantriol, Figure S2B, change of temperature did not result in change of emission vs pH dependence. These experiments clearly confirm that change of response pattern is due to change of structure of phytantriol nanoparticles.

The critical issue related to nanospheres optodes is their stability in dispersion.^29^ As it can be seen in Figure S3 for both high and low temperatures of the cubosome optodes, performance was not affected, within the range of experimental error, by storing for 45 days, which is a clear advantage of the herein proposed type of sensors. At a temperature of both 20 and 60 °C, cubosome optodes were highly selective; in the presence of model interferents–sodium or potassium chloride–no emission changes were observed, Figure S4A.

Due to its structure leading to the positioning of dye in the system,^9^ cubosome optodes also offer decreased bleaching of the emission signal in time.^30^ As it is shown in Figure S5, regardless if the probes were tested at 20 or 60 °C, both intensity at maximum and the response patterns characteristic for open/closed channels probes were preserved for at least ca. 210 min. This effect proves that not only cubosomes are offering stable analytical performance but also the structure of nanoprobe as such is stable in time, at both temperatures.

### Ca^2+^-Selective Lipid Based Nanostructural Optodes

**Figure 3:**
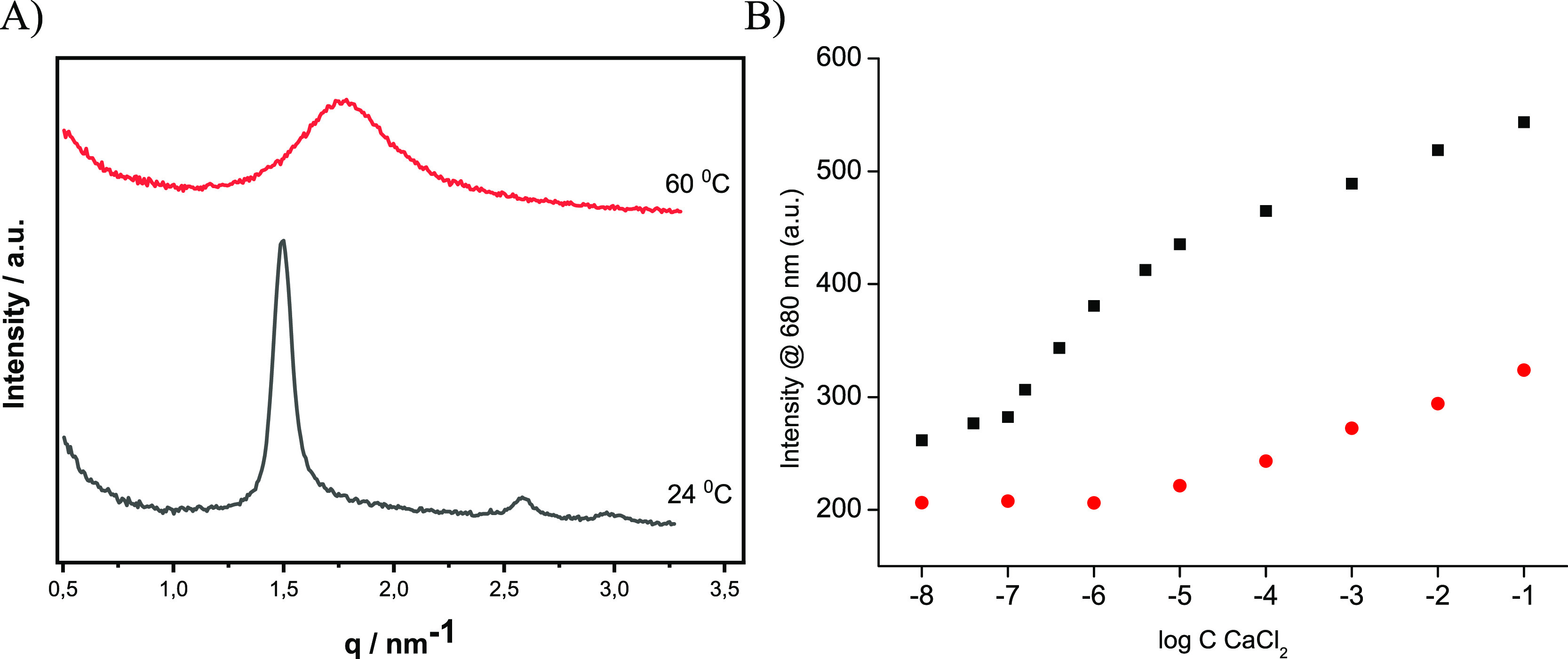
A) Representative SAXS diffraction patterns obtained for Ca^2+^-selective lipid based optodes at 24 and 60 °C. B) Emission at maximum at 680 nm dependence on CaCl_2_ concentration recorded for (■) 20 °C or (●, red) 60 °C, in the presence of 10^–2^ M Tris-HCl buffer, pH 7.2.

Figure 3 shows representative SAXS diffraction patterns obtained for sensor containing Ca^2+^-selective lipid based optodes. Introduction of a calcium-selective ionophore to the hydrogen-selective system to obtain Ca^2+^-selective optodes resulted in a change of the SAXS pattern recorded. As it can be seen in Figure 3, the SAXS spectra recorded display reflections spaced at √1, √3, and √4; the positioning of the Bragg peaks suggested the presence of the H_2_ phase–hexosomes. The presence of calcium ionophore (at the concentration used), a chromoionophore, and an ion exchanger promotes the formation of the H_2_ phase at a lower temperature than was observed for hydrogen sensors, Figure 1B.

The emission spectra recorded for Ca^2+^-selective optodes were similar to those shown in Figure 2A and 2B, with maximum emission formed for increasing analyte concentrations at 680 nm, Figure S6. For Ca^2+^-selective hexosomes, a closed channel system at 20 °C, linear dependence of emission signals at maximum 680 nm on logarithm of calcium ion concentration changes in solution is expected. Indeed, as shown in Figure 3B, a linear dependence of emission on logarithm of Ca^2+^ ion concentration in solution was obtained within the concentration range from 10^–5.4^ to 0.1 M (*R*^2^ = 0.992). Interestingly, for the concentration range from 10^–7^ to 10^–6^ M, the recorded signal was also linearly dependent on logarithm of concentration of CaCl_2_ in solution (*R*^2^ = 0.998), yet the slope of this part of dependence was much higher (3 times) compared to that observed for the higher concentration range. This is a unique effect, not reported earlier for other types of optodes or other structures/nanostructures. Taking into account that hexosomes are characterized with a closed channel structure at 20 °C, the observed linear emission changes for logarithm of concentration range from 10^–7^ to 10^–6^ M and can be attributed to gradual–sample concentration limited–saturation of the surface of the nanostructure. Postsaturation of the surface, the transport of analyte ions within the bulk of the probe is the rate limiting step similar to that previously reported for other types of nanostructural optodes, e.g., refs (6 and 9).

The temperature increase of Ca^2+^-selective optodes resulted in a change of the SAXS pattern, Figure 3A. After increasing the temperature to 60 °C, a broad diffuse peak was recorded on the SAXS spectrum, indicative of the L_2_ (reverse micellar) phase. The change of the structure of Ca^2+^-selective nanoprobes to reversed micelles, at 60 °C, resulted in a linear dependence of emission on logarithm of concentration within the range from 10^–6^ M to 10^–1^ M (*R*^2^ = 0.992), similar to other micellar type optodes.^31^ However, for concentration lower than 10^–6^ M, emission was not related to concentration change. Thus, the low detection limit observed at 20 °C, high sensitivity for change in analyte concentration from 10^–6^ to 10^–7^ M, is an important property of hexosomes, being clear proof of the unique advantages of lipid structures when used as optodes. It should be noted, that, under the same experimental conditions, similar to H^+^-selective optodes, higher emission intensities were observed at lower temperatures. At temperatures of both 20 and 60 °C, Ca^2+^-selective cubosome optodes were highly selective; in the presence of a model interferent–sodium chloride–no emission changes were observed, Figure S4B.

The results presented in Figure 3B clearly show that lipid nanostructures, prepared using a biocompatible matrix, offer unique analytical parameters of ion-selective optodes.

## Conclusions

Novel type optical sensors are proposed–cubosome or hexosome optodes. The nanostructures prepared from the biocompatible matrix allow elimination of a toxic plasticizer offering sensors of high and tunable sensitivity and stability in dispersion and in a few hours’ time signal. The unique feature of cubosome/hexosome optodes is the possibility to increase sensitivity by a temperature trigger leading to spontaneous rearrangement of the internal structure of the probes. Clearly it is rational to expect that for cubosome optodes, structural and analytical properties, in general, will be related to structure and properties of the ionophore and lipid used; however, a broad family of lipid molecules that can be applied to prepare cubosomes offers the possibility to prepare many different nanoparticle ion-selective optical sensors.

Due to unique properties herein, proposed optodes are potentially attractive not only for applications in contact with living organisms but also for other applications.

## Supporting Information Available

Absorbance changes of cubosome probes, dependence of fluorescence intensity on pH, signal vs pH recorded for temperature change from 20 to 60 °C, same dependence recorded for 45 day old spheres, dependence of fluorescence intensity vs pH recorded at 60 or 20 °C for model interferents studied, dependence of fluorescence intensity vs pH recorded at 60 or 20 °C for control lipid structures from monoolein, emission vs pH dependence recorded after 180 or 210 min contact of probes with sample, and emission spectra of Ca^2+^-selective optodes recorded at 20 and 60 °C for different concentrations of analyte (PDF)

## References

[ref1] Ruedas-Rama M. J.; Walters J. D.; Orte A.; Hall E. A. H. Fluorescent nanoparticles for intracellular sensing: a review. Anal. Chim. Acta 2012, 751, 1–23. 10.1016/j.aca.2012.09.025. 23084048

[ref2] Morf W. E.; Seiler K.; Rusterholz B.; Simon W. Design of a Calcium-Selective Optode Membrane Based on Neutral Ionophores. Anal. Chem. 1990, 62, 738–742. 10.1021/ac00206a018. 1868594

[ref3] Tsagkatakis I.; Peper S.; Bakker E. Spatial and Spectral Imaging of Single Micrometer-Sized Solvent Cast Fluorescent Plasticized Poly(vinyl chloride) Sensing Particles. Anal. Chem. 2001, 73, 315–320. 10.1021/ac000832f. 11199984

[ref4] Kisiel A.; Kłucińska K.; Głębicka Z.; Gniadek M.; Maksymiuk K.; Michalska A. Alternating polymer micelle nanospheres for optical sensing. Analyst 2014, 139, 2515–2524. 10.1039/c3an02344c. 24665466

[ref5] Ruedas-Rama M. J.; Hall E. A. H. K^+^-selective nanospheres: maximising response range and minimising response time. Analyst 2006, 131, 1282–1291. 10.1039/b608901a. 17124535

[ref6] Woźnica E.; Maksymiuk K.; Michalska A. Polyacrylate Microspheres for Tunable Fluorimetric Zinc Ions Sensor. Anal. Chem. 2014, 86, 411–418. 10.1021/ac4033142. 24294919

[ref7] Espadas-Torre C.; Meyerhoff M. E. Thrombogenic Properties of Untreated and Poly(ethylene oxide)-Modified Polymeric Matrixes Useful for Preparing Intraarterial Ion-Selective Electrodes. Anal. Chem. 1995, 67, 3108–3114. 10.1021/ac00114a003. 8686883

[ref8] Lindner E.; Cosofret V. V.; Ufer S.; Buck R. P.; Kao W. J.; Neuman M. R.; Anderson J. M. Ion-selective membranes with low plasticizer content: electroanalytical characterization and biocompatibility studies. J. Biomed. Mater. Res. 1994, 28, 591–601. 10.1002/jbm.820280509. 8027099

[ref9] Stelmach E.; Kłucińska K.; Maksymiuk K.; Michalska A. Rational design of nanoptodes architecture – Towards multifunctional sensors. Talanta 2019, 196, 226–230. 10.1016/j.talanta.2018.12.047. 30683356

[ref10] Xie X.; Mistlberger G.; Bakker E. Ultrasmall Fluorescent Ion-Exchanging Nanospheres Containing Selective Ionophores. Anal. Chem. 2013, 85, 9932–9938. 10.1021/ac402564m. 24020858

[ref11] Baranowska-Korczyc A.; Maksymiuk K.; Michalska A. Electrospun nanofiber supported optodes: scaling down the receptor layer thickness to nanometers – towards 2D optodes. Analyst 2019, 144, 4667–4676. 10.1039/C9AN00756C. 31265013

[ref12] Kisiel A.; Kałuża D.; Paterczyk B.; Maksymiuk K.; Michalska A. Quantifying plasticizer leakage from ion-selective membranes – a nanosponge approach. Analyst 2020, 145, 2966–2974. 10.1039/C9AN02621E. 32115595

[ref13] Alcaraz N.; Boyd B. J. Cubosomes as Carriers for MRI Contrast Agents. Curr. Med. Chem. 2017, 24, 470–482. 10.2174/0929867323666160817141556. 27538694

[ref14] Meli V.; Caltagirone C.; Sinico C.; Lai F.; Falchi A. M.; Monduzzi M.; Obiols-Rabasa M.; Picci G.; Rosa A.; Schmidt J.; Talmon Y.; Murgia S. Theranostic hexosomes for cancer treatments: an *in vitro* study. New J. Chem. 2017, 41, 1558–1565. 10.1039/C6NJ03232J.

[ref15] Nazaruk E.; Szlęzak M.; Górecka E.; Bilewicz R.; Osornio Y. M.; Uebelhart P.; Landau E. M. Design and Assembly of pH-Sensitive Lipidic Cubic Phase Matrices for Drug Release. Langmuir 2014, 30, 1383–1390. 10.1021/la403694e. 24443890

[ref16] Azhari H.; Strauss M.; Hook S.; Boyd B. J.; Rizwan S. B. Stabilising cubosomes with Tween 80 as a step towards targeting lipid nanocarriers to the blood–brain barrier. Eur. J. Pharm. Biopharm. 2016, 104, 148–155. 10.1016/j.ejpb.2016.05.001. 27163239

[ref17] Landau E. M.; Rosenbusch J. P. Lipidic cubic phases: A novel concept for the crystallization of membrane proteins. Proc. Natl. Acad. Sci. U. S. A. 1996, 93, 14532–14535. 10.1073/pnas.93.25.14532. 8962086 PMC26167

[ref18] Cherezov V.; Clogston J.; Papiz M. Z.; Caffrey M. Room to Move: Crystallizing Membrane Proteins in Swollen Lipidic Mesophases. J. Mol. Biol. 2006, 357, 1605–1618. 10.1016/j.jmb.2006.01.049. 16490208

[ref19] Final Report on the Safety Assessment of Phytantriol. Int. J. Toxicol. 2007, 26 ( (Suppl. 1), ), 107–114, 10.1080/10915810601163947. 17365138

[ref20] Barriga H. M. G.; Holme M. N.; Stevens M. M. Cubosomes:The Next Generation of Smart Lipid Nanoparticles?. Angew. Chem., Int. Ed. 2019, 58, 2958–2978. 10.1002/anie.201804067. PMC6606436 29926520

[ref21] Dailey A. L.; Greer M. D.; Sodia T. Z.; Jewell M. P.; Kalin T. A.; Cash K. J. LipiSensors: Exploiting Lipid Nanoemulsions to Fabricate Ionophore-Based Nanosensors. Biosensors 2020, 10, 120–132. 10.3390/bios10090120. PMC7557773 32927619

[ref22] Rowinski P.; Rowinska M.; Heller A. Liquid crystal membranes for serum-compatible diabetes management-assisting subcutaneously implanted amperometric glucose sensors. Anal. Chem. 2008, 80, 1746–1755. 10.1021/ac702151u. 18247485

[ref23] Murgia S.; Biffi S.; Mezzenga R. Recent advances of non-lamellar lyotropic liquid crystalline nanoparticles in nanomedicine. Curr. Opin. Colloid Interface Sci. 2020, 48, 28–39. 10.1016/j.cocis.2020.03.006.

[ref24] Mertins O.; Mathews P. D.; Angelova A. Advances in the Design of pH-Sensitive Cubosome Liquid Crystalline Nanocarriers for Drug Delivery Applications. Nanomaterials 2020, 10, 963 10.3390/nano10050963. PMC7281514 32443582

[ref25] Barauskas J.; Landh T. Phase Behavior of the Phytantriol/Water System. Langmuir 2003, 19, 9562–9565. 10.1021/la0350812.

[ref26] Alvarez-Malmagro J.; Matyszewska D.; Nazaruk E.; Szwedziak P.; Bilewicz R. PM-IRRAS Study on the Effect of Phytantriol-Based Cubosomes on DMPC Bilayers as Model Lipid Membranes. Langmuir 2019, 35 (50), 16650–16660. 10.1021/acs.langmuir.9b02974. 31746606

[ref27] Alvarez-Malmagro J.; Jablonowska E.; Nazaruk E.; Szwedziak P.; Bilewicz R. How do lipid nanocarriers – Cubosomes affect electrochemical properties of DMPC bilayers deposited on gold (111) electrodes?. Bioelectrochemistry 2020, 134, 107516 10.1016/j.bioelechem.2020.107516. 32222670

[ref28] Sagalowicz L.; Mezzenga R.; Leser M. E. Investigating reversed liquid crystalline mesophases. Curr. Opin. Colloid Interface Sci. 2006, 11, 224–229. 10.1016/j.cocis.2006.07.002.

[ref29] Woźnica E.; Gasik J.; Kłucińska K.; Kisiel A.; Maksymiuk K.; Michalska A. Core-shell nanoparticles optical sensors - Rational design of zinc ions fluorescent nanoprobes of improved analytical performance. Opt. Mater. 2017, 72, 214–219. 10.1016/j.optmat.2017.05.059.

[ref30] Langmaier J.; Lindner E. Detrimental changes in the composition of hydrogen ion-selective electrode and optode membranes. Anal. Chim. Acta 2005, 543, 156–166. 10.1016/j.aca.2005.04.011.

[ref31] Kisiel A.; Kłucińska K.; Głębicka Z.; Gniadek M.; Maksymiuk K.; Michalska A. Alternating polymer micelle nanospheres for optical sensing. Analyst 2014, 139, 2515–2524. 10.1039/c3an02344c. 24665466

