# Supplementary material for: Cubosome Based Ion-Selective Optodes–Toward Tunable Biocompatible Sensors
Source: Anal Chem. 2021 Sep 21;93(39):13106–11. doi: 10.1021/acs.analchem.1c01247 (PMC8495674; doi:10.1021/acs.analchem.1c01247)
Supplement: Supplementary file 1 — ac1c01247_si_001.pdf [file ac1c01247_si_001.pdf]

Supporting information

Cubosome based ion-selective optodes – towards tunable biocompatible sensors

Emilia Stelmach, Ewa Nazaruk, Krzysztof Maksymiuk, Agata Michalska*

Faculty of Chemistry, University of Warsaw, Pasteura 1, 02-093 Warsaw, Poland

* agatam@chem.uw.edu.pl, +48 22 56 22 331

Table of Content

1. Absorbance changes of cubosome probes in (blue line) 0.1 M NaOH and (red line) in 0.1 M HCl.

2. Dependence of fluorescence intensity on pH, the signal vs. pH recorded for temperature change from 20 °C to 60 °C,

3. Dependence of fluorescence intensity on pH, the signal vs. pH recorded for temperature change from 20 °C to 60 °C recorded for 45 days old spheres.

4. Dependence of fluorescence intensity read at maximum 680 nm recorded at 60 °C or 20 °C on changes of logarithm of concentration of model interferents.

5. Dependence of fluorescence intensity on pH, the mean signal recorded at 680 nm recorded at 20 °C or 60 °C after longer contact time of probes with the sample.

6. Effect of Ca^{2+} ions concentration change on emission of cubosome optodes: emission spectra recorded at 20 °C and 60 °C.

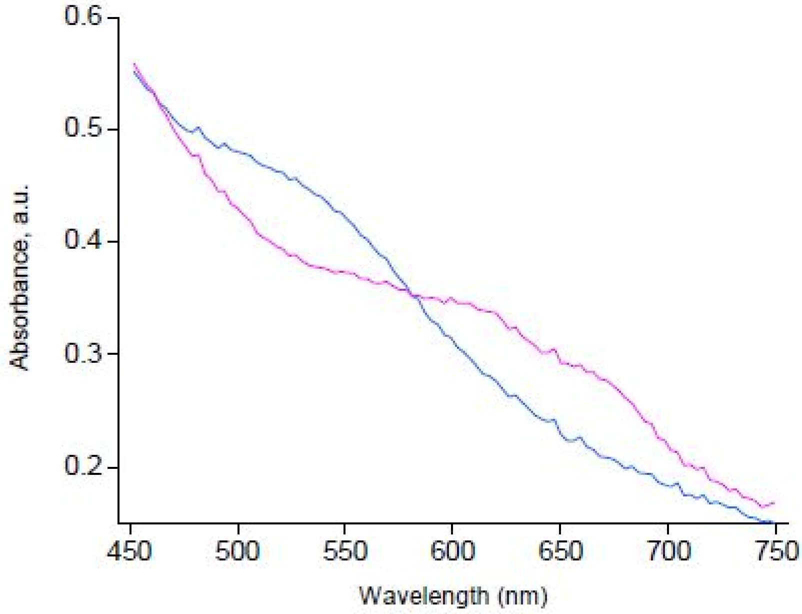

Figure S1. Absorbance changes of cubosome probes in (blue line) 0.1 M NaOH and (red line) in 0.1 M HCl.

A)

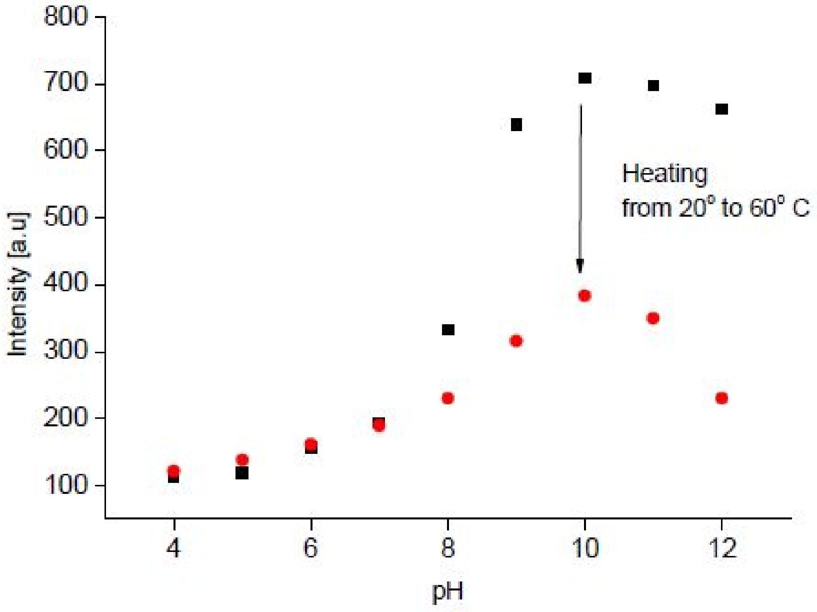

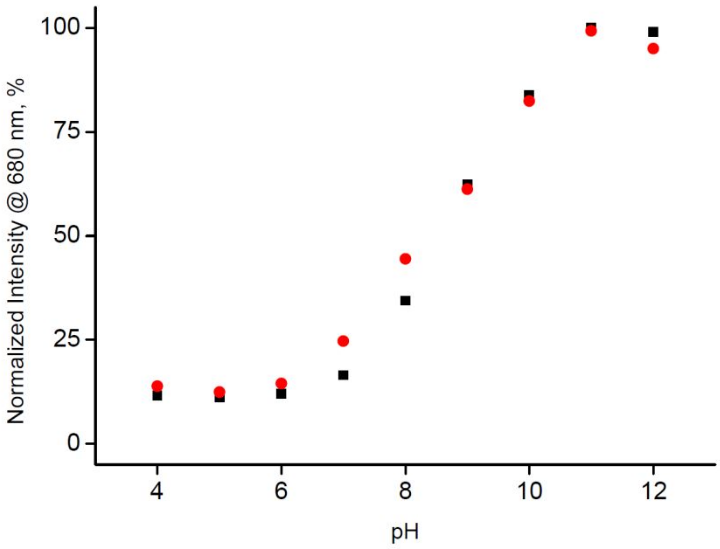

B)

Figure S2. A) Dependence of fluorescence intensity on pH, the signal recorded at 680 nm recorded for temperature change from (■) 20°C to (●) 60°C for phytantriol nanoparticles. B) Emission read at 680 nm plotted as function of pH recorded for monoolein nanoparticles at (■) 20°C and (●) 60°C.

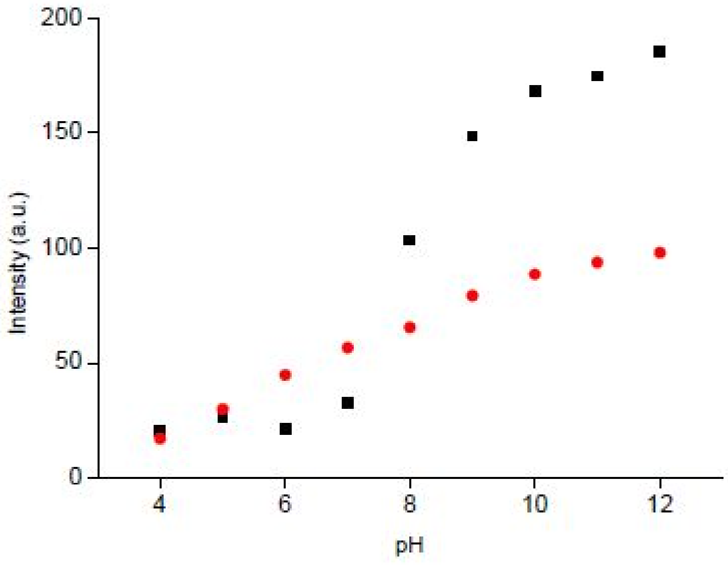

Figure S3. Dependence of fluorescence intensity on pH, the mean signal recorded at 680 nm recorded at (■) 20°C or (●) 60°C for 45 days old spheres.

A)

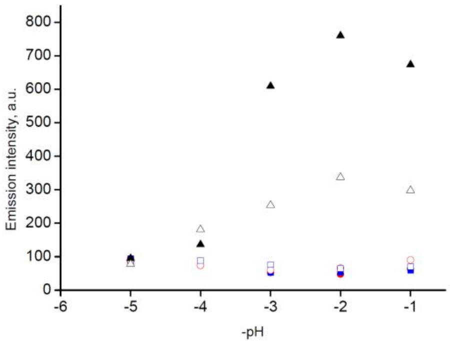

B)

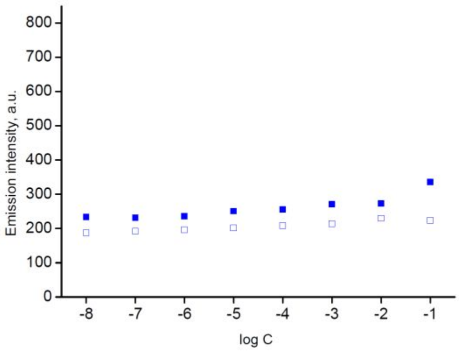

Figure S4. Dependence of fluorescence intensity read at maximum 680 nm recorded at (open symbols) 60°C or (closed symbols) 20°C on: A) pH for H^+-selective lipid based nanostructural optodes (●/○) KCl, (■/□) NaCl or (▲/Δ) NaOH and B) logarithm of concentration for Ca^{2+}-selective lipid based nanostructural optodes (■/□) NaCl.

A)

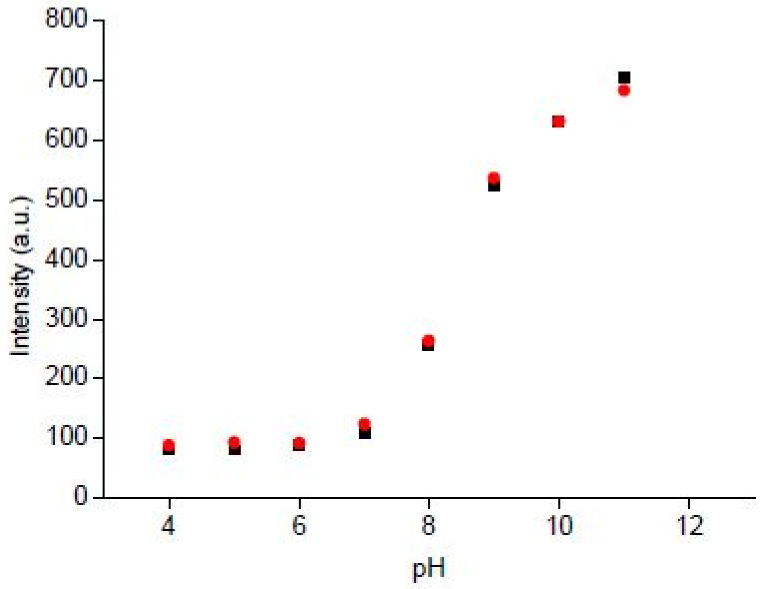

B)

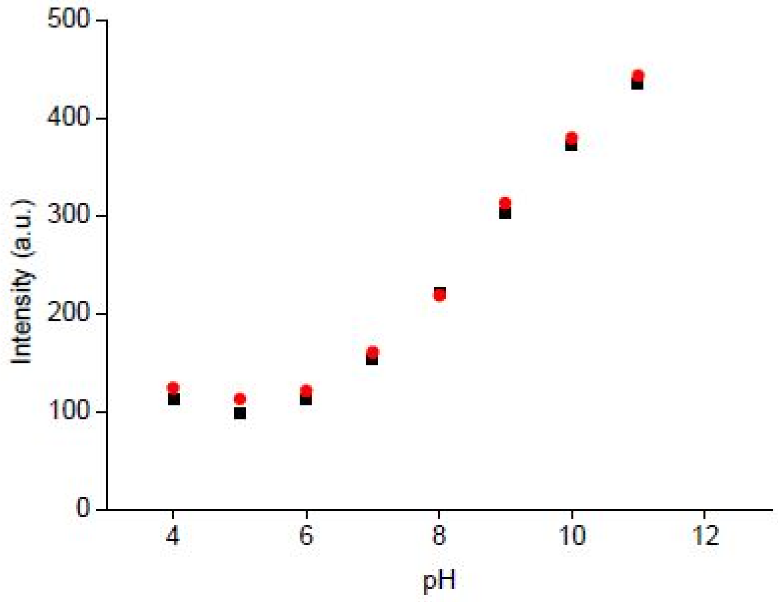

Figure S5. Dependence of fluorescence intensity on pH, the mean signal recorded at 680 nm recorded at A) 20°C or B) 60°C after (■) 180 or (●) 210 minutes contact with the sample.

A)

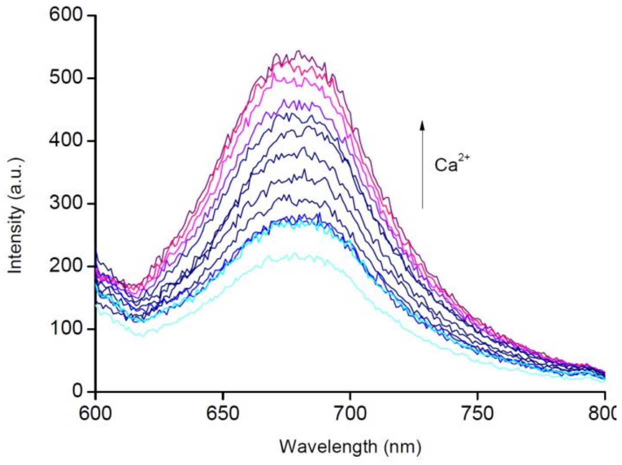

B)

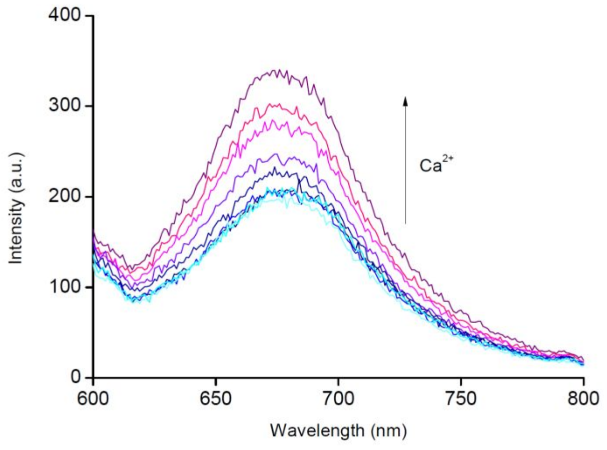

Figure S6. Effect of Ca^{2+} ions concentration change on emission of cubosome optodes: emission spectra recorded at A) 20°C and B) 60°C.
